# Inductive Microsensor for Magnetic Field Detection: Application in Wireless Power Transfer Systems

**DOI:** 10.3390/s26175382

**Published:** 2026-08-26

**Authors:** Teth Azrael Cortes-Aguilar, Ruth Yadira Vidaña-Morales, David Gómez-Gutiérrez, Daniel Rafael Vidaña-Morales

**Affiliations:** Departamento de Ingeniería Electrónica, Tecnológico Nacional de México, ITJMMPyH, Campus Zapopan, Zapopan 45100, Jalisco, Mexico; teth.cortes@zapopan.tecmm.edu.mx (T.A.C.-A.); za250120013@zapopan.tecmm.edu.mx (D.R.V.-M.)

**Keywords:** MEMS, induction microsensor, leakage magnetic field, wireless power transmission (WPT)

## Abstract

**Highlights:**

Cost-Effective Sensor Design: Utilizes photolithography to fabricate a low-cost multi-layer MEMS sensor on a silicon substrate with Ni/Cr metallic layers.
Real-World Application: Enhances system safety in wireless power transmission by monitoring stray magnetic fields to avoid unintended heating of conductive parts.
Advanced coil Fabrication: A compact inductive microsensor featuring a versatile architecture for integration into portable magnetic field diagnostic instruments.
Future Trends: Enables precise spatial mapping of magnetic leakage via an induction microsensor to ensure electromagnetic compatibility in WPT systems.

**Abstract:**

Wireless Power Transfer (WPT) has emerged as a compelling alternative to wired charging; however, efficiency and safety are highly dependent on magnetic field distribution and leakage. This work presents a compact MEMS–based magnetic field induction sensor, including its design, fabrication, and electrical characterization, for real-time diagnosis in WPT systems. The sensor employs a Ni/Cr metallic inductor fabricated on a SiO_2_ substrate using standard photolithography and occupies a footprint of 5 mm × 5 mm. The device is electrically characterized through impedance, quality factor, and frequency response measurements, followed by experimental validation using an industry-standard wireless charging system and dedicated signal-conditioning circuitry. The results from inductive coupling simulations, performed with the Magpylib Python library, align with experimental data showing that the sensor accurately follows theoretical magnetic field decay, detecting AC signals between 40 mV and 140 mV with a functional limit of 30 mm. Furthermore, experimental characterization through spatial mapping successfully identifies magnetic leakage hot spots, while thermal validation via infrared thermography correlates these magnetic readings with localized temperature increases. This integrated approach supports EMC optimization and thermal risk mitigation, providing a low–cost and effective diagnostic tool for enhancing safety and performance in WPT applications.

## 1. Introduction

Magnetic field coupling is a form of Wireless Power Transfer (WPT) that utilizes magnetic fields to transfer energy from a transmitter (Tx) to a receiver (Rx) [1]. The Tx acts as the energy source by converting electrical power from a supply into a high-frequency alternating current, which flows through a primary coil to generate a time-varying magnetic field. This field bridges the air gap to the Rx, where the magnetic flux penetrates a secondary coil and induces an alternating current through Faraday’s Law of Induction. The energy captured by the receiver is rectified and filtered to provide a stable DC output, suitable for charging a battery or powering an electronic device. This technology is generally categorized into two types: inductive wireless power transfer and magnetic resonant coupling [2].

While both technologies utilize magnetic fields for energy transmission, the primary difference lies in their coupling mechanism and operational range. Inductive WPT operates like an air-core transformer, relying on a high coupling coefficient between two coils placed in very close proximity, generally within a range of a few millimeters; it is highly efficient but extremely sensitive to misalignment. In contrast, Magnetic Resonant Coupling employs a pair of resonators synchronized to an identical frequency, which enables the efficient transfer of energy across mid-range distances spanning from centimeters to meters.

Inductive WPT is a simple, well-established technology already widely adopted in the consumer market for smartphones [3] and smartwatches [4]. Conversely, magnetic resonant coupling is a more sophisticated, emerging technology for charging electric vehicles (EVs) [5,6,7]. By eliminating the physical connectors required by traditional plug-in systems, it significantly improves user safety and operational efficiency.

Within the automotive Electric Vehicle (EV) sector, the SAE J2954 standard [8], launched in 2020, provides an industry-wide specification for WPT, defining critical criteria for interoperability, alignment tolerance, and electromagnetic compatibility. Furthermore, it establishes benchmarks for minimum performance and rigorous safety limits for electromagnetic field (EMF) exposure. Similarly, for consumer electronics, the Wireless Power Consortium [9] has standardized low-power applications, beginning with the Qi 1.0 launch in 2010. While that initial 5 W version focused on safety features like Foreign Object Detection (FOD) to prevent metallic overheating, the latest Qi 2.2 standard, released in 2025, utilizes magnetic alignment to optimize energy transfer and supports power levels up to 25 W. Consequently, WPT has emerged as a transformative alternative to traditional conductive charging, delivering superior convenience, safety, and user experience across a spectrum ranging from low-power consumer electronics to high-power industrial applications.

Despite recent advancements, WPT technology continues to face challenges regarding cost-efficiency, foreign object detection, air gap constraints, high sensitivity to misalignment, and inherent transfer losses [10,11]. However, magnetic leakage fields raise significant concerns regarding electromagnetic interference with nearby electronics and the challenge of complying with EMF safety standards. Detecting this leakage is critical to ensuring both the safety and the overall performance of WPT systems [12].

**Figure 1:**
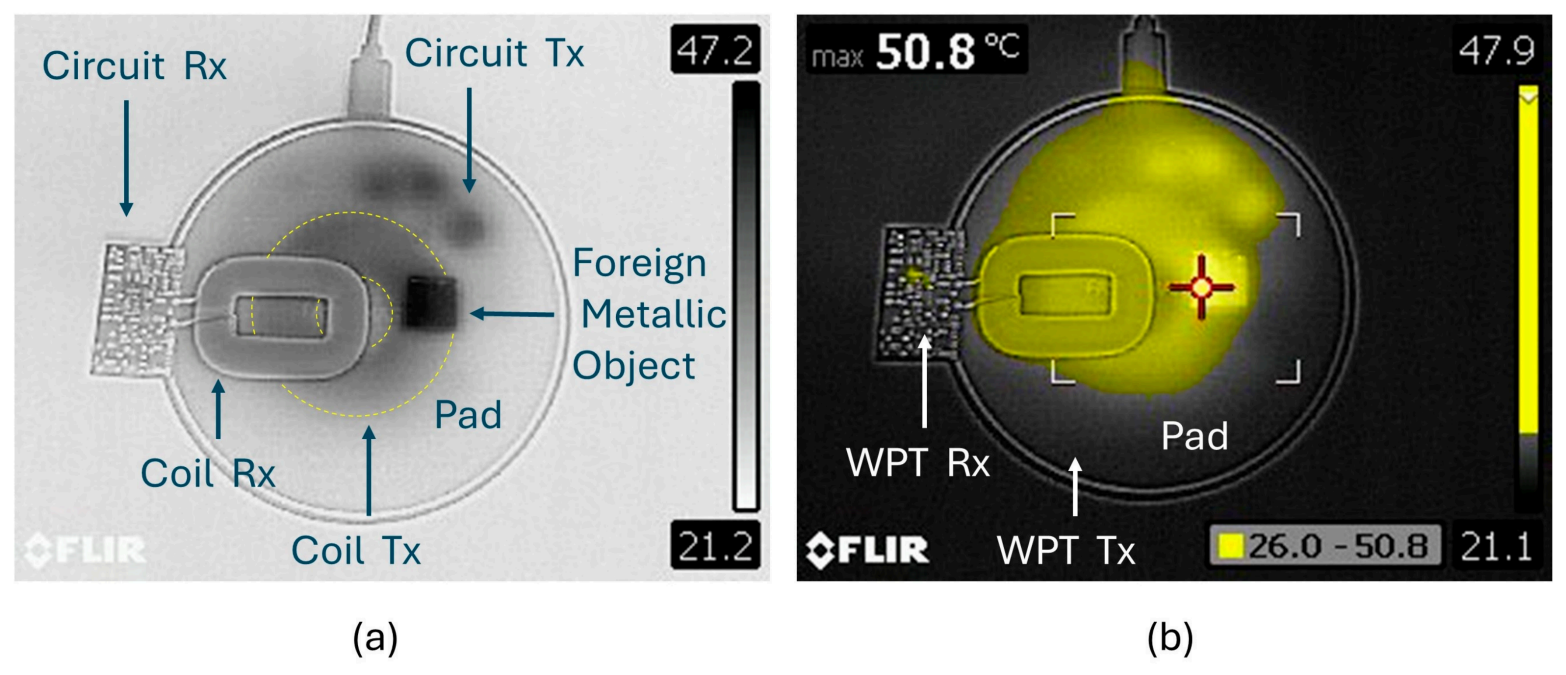
Thermal analysis of a wireless power transfer system (**a**) Foreign metallic object placed on the Tx pad; (**b**) Thermal distribution and peak temperature measurement of the foreign object.

Figure 1 presents a thermal analysis of a Qi WPT system. As shown in Figure 1a, a foreign metallic object is placed on the Tx pad, directly above the transmitter coil. This object consists of a square aluminum film with a black vinyl cover; the cover provides a high emissivity of 0.95 to ensure accurate temperature measurements. Figure 1b demonstrates that the object reaches a peak temperature of 50.8 °C only 30 s after the transmitter is activated. Such rapid heating represents a critical safety issue that could damage the system or even cause burns to the devices or the user. The thermograms in Figure 1 were captured using a FLIR E50 handheld thermal camera (Teledyne FLIR LLC, Wilsonville, OR, USA).

While several instruments are available for measuring magnetic fields, the development of compact, low-cost sensors specifically designed to detect magnetic leakage and transmitter malfunctions represents a significant contribution to enhancing the performance and safety of WPT systems. The sensitivity, comparative advantages, and typical applications of different sensor technologies are summarized in Table 1.

In contrast to conventional sensors, MEMS technology offers inherent advantages such as small size, lightweight design, and feasible integration. Furthermore, their compatibility with batch fabrication ensures cost–effectiveness, leading to their widespread adoption across the consumer electronics and automotive sector.

Several previous studies have addressed the development of miniaturized magnetic sensors and thin-film inductive devices. For instance, Gatzen et al. [26] reported an eddy–current microsensor fabricated using thin-film technology, including a soft-magnetic core and separated excitation and pickup coils, with an operating frequency range from 100 kHz to 2 MHz. However, their device was mainly intended for proximity measurement and nondestructive evaluation of conductive targets, such as surface contour analysis and microcrack detection. In contrast, the present work does not use the sensor as an active eddy-current probe for material inspection; instead, it employs a passive Ni/Cr thin-film inductive coil to directly detect the alternating magnetic leakage field generated by a commercial wireless power transfer transmitter operating at 132.7 kHz. Other MEMS–based magnetic field mapping approaches, such as Hall sensor arrays [27], have demonstrated two-dimensional magnetic field reconstruction, but they are generally based on semiconductor Hall–effect sensing and are not specifically designed for high-frequency inductive WPT leakage diagnosis. Similarly, sensor or shielding coils used in WPT systems [10] are mainly integrated into the power transfer structure to detect receiver misalignment or reduce leakage fields. Therefore, the main difference in this work is its application-oriented design: a compact, low–cost, thin-film inductive microsensor combined with portable signal conditioning for quantitative spatial mapping of magnetic leakage hot spots and thermal-risk regions in consumer-grade WPT systems.

The main contributions of this paper are as follows. First, a compact MEMS–based inductive magnetic field sensor with a footprint of 5 mm × 5 mm was designed and fabricated using a Ni/Cr thin-film coil compatible with standard photolithographic processing. Second, the sensor was experimentally characterized at the operating frequency of a commercial Qi wireless charger, where it detected AC signals in the range of approximately 40–140 mV and showed a functional sensing distance of up to 30 mm. Third, a portable single-supply interface circuit was developed to amplify the induced high–frequency AC signal and convert it into a stable DC output suitable for LED indication or microcontroller-based acquisition. Fourth, the sensor was validated through quantitative spatial mapping of magnetic leakage in a commercial WPT system, enabling the identification of localized magnetic hot spots near the receiver coil and associated circuitry. Finally, thermal imaging was used to correlate the measured magnetic leakage regions with localized temperature increases, demonstrating the practical value of the proposed sensor for WPT diagnostics, electromagnetic compatibility assessment, foreign-object risk analysis, and thermal safety evaluation.

This paper is organized as follows. Section 2, Related Works, reviews various materials and methodologies currently utilized in the fabrication of MEMS sensors. Section 3, Materials and Methods, details the design and fabrication of the proposed inductive MEMS sensor, alongside the interface circuitry developed to amplify signals for single-source DC portable instruments. Section 4, Results, presents the sensor’s normalized output voltage relative to its vertical separation from a commercial transmitter coil. This section also evaluates the output waveforms and the signal conditioning performance of the interface circuit, concluding with an experimental analysis of magnetic leakage within a commercial Qi system. Section 5 provides a Discussion on the sensor’s effectiveness for spatial mapping, its potential applications, and future research directions. Finally, Section 6 presents the conclusions of this work.

## 2. Related Works

This section examines diverse materials and methods used to fabricate MEMS sensors, ranging from micro–fluxgate designs with Co-based alloys to Hall–effect arrays on silicon substrates. It details the use of photolithography and High-Density Plasma Chemical Vapor Deposition for layering dielectric materials like silicon oxide. The text also covers specialized techniques such as depositing piezoelectric Aluminum Nitride (AIN) thin films and magnetostrictive multilayers onto silicon wafers. Furthermore, it explores CMOS–compatible processes involving vapor HF etching to release movable structures. These methods emphasize the engineering balance between sensor sensitivity and the complexity of the interface circuitry required to maintain signal integrity.

High–precision MEMS magnetometers are essential for numerous applications and are categorized as micro-fluxgate, Hall effect, and magnetoresistive sensors. The primary challenges in developing micro-fluxgate sensors lie in the precision miniaturization of solenoid coils and the integration of a magnetic core. Hall–effect magnetic sensors are widely adopted due to their full compatibility with cost-effective CMOS semiconductor processes. However, they generally exhibit lower sensitivity when compared to alternative MEMS magnetometer technologies. Anisotropic Magneto–Resistive (AMR) sensors leverage the magnetoresistive effect in ferromagnetic films to achieve high sensitivity and cost-efficiency. Despite these advantages, they often require complex reset mechanisms due to magnetic saturation occurring at the mT level [28].

The authors in [29] used MEMS processes to deposit a 2 μm thick piezoelectric AIN thin film onto a 350 μm thick silicon wafer. The magnetostrictive multilayer was built up by an eightfold repetition of Ta, Cu, Mn_80_Ir_20_, and FeCoSiB with thicknesses of 8 nm, 10 nm, 3 nm, and 500 nm, respectively. This sensor was designed to exhibit antiparallel magnetization between adjacent layers. Following the deposition of the functional thin films, the wafer was diced into cantilever structures with dimensions of 24.95 mm by 2.45 mm. The cantilever was mounted on a carrier board and integrated into a pickup coil consisting of 800 turns of 100 µm thick enameled copper wire. An electrical excitation in the cantilever creates an oscillating stress in the magnetostrictive layer, which leads to an oscillating response of the magnetization. This oscillation is detected inductively via a pickup coil; the resulting signal is transmitted to a low-noise operational amplifier with a gain of 10 and subsequently to a high-speed lock-in amplifier for characterization.

In [30], a fluxgate sensor was fabricated using standard MEMS processing. The sensor contains a rectangular magnetic core–composed of an amorphous Co–based soft magnetic alloy–wrapped in two pairs of driving coils. The overall sensor package measures approximately 12 mm by 7 mm. Specifically, the magnetic core dimensions are 11.05 mm by 2.35 mm with a thickness of 15 μm, while the width of the long-side segments is 650 μm. To capture signal variations induced by magnetic flux, a 59–turn pick-up coil was integrated at the center of the core, flanked by two 62–turn driving coils. According to the authors, the sensor exhibits an excellent capability for detecting weak magnetic fields compared to other designs, as its sensitivity can be enhanced by increasing the driving frequency. However, they noted that this approach increases circuit complexity and power consumption. Furthermore, the voltage noise spectral density must be reduced to optimize the sensor’s overall resolution.

In [31], the authors reported a novel technology for the creation of a magnetoelectric (ME) MEMS sensor based on a b-LN/FE_70_CO_8_Si_12_B_10_ heterostructure comprising bidomain lithium niobate (b-LN) and Metglas layers. The magnetostrictive layer has a thickness of 2 μm, and the b-LN cantilever is 80 μm thick and 4.5 mm by 3 mm in size. Additionally, a 100 nm–thick Nichrome Cr_20_Ni_80_ contact was deposited on the opposite side of the cantilever. According to the authors, the b-LN/FE_70_CO_8_Si_12_B_10_ laminate sensor has a magnetoelectric coefficient comparable to that of AIN-based structures and exhibits the highest conversion coefficient. Nevertheless, signals gathered by ME MEMS sensors are inherently noisy; therefore, additional measures for data collection and processing, such as active shielding, digital filtering, noise cancelation, and adaptive averaging, must be applied. This sensor, integrated with a high-resolution Analog–to–Digital Converter (ADC) and sophisticated signal processing, was employed to detect biomagnetic signals from the human heart.

In [27], the authors fabricated an array of Hall sensors on silicon substrates using standard MEMS fabrication techniques. Four Hall sensors featuring detection zones with identical aspect ratios but varying sizes were manufactured. The dimensions were 2 mm by 0.32 mm for the small sensor, 3 mm by 0.48 mm for medium, 4 mm by 0.64 mm for large, and 5 mm by 0.8 mm for extra–large. A silicon oxide isolation layer with a thickness of 0.5 µm was deposited on the surface of the 4-inch P-type Si wafer (Thickness: 525 +/− 25 µm, Internal Resistivity: 1–20 Ω–cm) using a High-Density Plasma Chemical Vapor Deposition technique. A photolithography method was employed to pattern and etch the cross-shaped detection zone in the Si substrate, and the detection zone was then implanted with phosphide ions. The Electron Beam Evaporation method was used to pattern the sensor surface with AU/Cr for electrical connection purposes. The authors utilized this array to map the magnetic fields of both a single magnet and an arrangement of three magnets for nondestructive testing purposes.

The work in [32] describes various design techniques for MEMS fabrication within standard complementary metal-oxide semiconductor CMOS processes. These methods were applied specifically to the development of shielded Lorentz–force magnetometers. The authors utilized the back–end–of–line (BEOL) of a standard 6–metal 0.18 μm CMOS process to fabricate the MEMS. The unwanted intermetal dielectric oxide is etched away with a vapor Hydrogen Fluoride (vHF) process. The vHF enters through small holes in the last metal of the BEOL, dissolving the oxide selectively and releasing the MEMS structure. For fabrication reasons, each BEOL layer consists of several sub-layers. Finally, the MEMS devices are sealed in vacuum with a post–CMOS layer of Aluminum deposited on top of the last metal, diced, and packaged. To sense the magnetic field, a current is passed through a wire of length within a movable structure. This generates a Lorentz force given by $\vec{F}=L\cdot \vec{I_{L}}\times \vec{B}$ that drives the system, which acts as a damped harmonic oscillator. A square-wave current was applied with a frequency tuned to the first resonant frequency of the structure, thereby maximizing the sensed current through resonant amplification. While the demagnetization effect, material permeability, and structural configuration significantly influence the performance of most MEMS sensors, the absence of magnetic materials in LFM provides a distinct advantage. However, significant offsets persist in these devices. To address this, a current chopping technique must be implemented alongside beam shielding to minimize electrical interference and improve signal integrity.

In [33], the authors fabricated magnetoelectric MEMS resonators with various dimensions ranging from 400 to 850 µm and widths from 60 to 125 µm. The individual resonators were based on electromechanical thin-film multilayer structures consisting of a 10 µm thick doped poly-Si substrate, which also functions as a rear-side electrode. A 0.2 µm thick pad oxide layer was included on top to provide electrical insulation from subsequent layers. On top of the pad oxide layer, a 0.5 µm thick AIN piezoelectric layer was deposited, followed by two 1 µm thick patterned Al electrodes symmetrically placed on both sides of the anchors for actuation and readout. To complete the structure, a 200 nm amorphous FeCoSiB magnetostrictive layer was deposited on the rear of the resonators. Large effective anisotropies or small magnetostriction reduce the influence of stress on the magnetic properties but simultaneously decrease the sensor’s sensitivity. Consequently, developing reliable fabrication techniques for magnetoelastic sensors is crucial to address these issues. Typically, these devices are fabricated by depositing and patterning a magnetoelastic layer on a constrained resonator, which is subsequently released. During this release process, however, anisotropic stress is unintentionally introduced into the magnetoelastic layer through the relaxation of intrinsic stresses within the substrate and underlying layers. As a result, the residual stress in the magnetic layer is not only determined by the magnetic layer deposition process but also by the fabrication of the other underlying layers and the substrate.

In [34] fabricated a micro-orthogonal fluxgate (OFG) sensor using an amorphous CoZrNb magnetic film. The sensor was 1.5 mm long with an effective region of 0.76 mm, suggesting its compatibility with compact multi–chip packaging. This study aimed to develop a micro magnetic sensor capable of detecting the Earth’s magnetic field. The prototype reached its maximum sensitivity at a 10 MHz operational frequency, producing a 9.8 mV output voltage at 9.98 Oe. The output voltage and sensitivity of the OFG sensor depend on the operating frequency, as well as the turn number of the detection coil and the cross-sectional area; it exhibits an upper frequency limit of ~10 MHz. Surpassing this threshold does not result in an enhancement of sensitivity. The magnetization of the sensor becomes saturated at a higher external magnetic field in the longitudinal direction. Additionally, as the conductor width decreases, the nonlinearity of the output near 0 Oe becomes more pronounced.

According to the International Commission on Non–Ionizing Radiation Protection (ICNIRP) guidelines, EMF exposure poses potential health risks, making safety compliance essential for WPT manufacturers. While nerve stimulation is the primary concern at frequencies between 3 kHz and 10 MHz, thermal effects become the more prominent risk as frequencies approach 10 MHz, potentially leading to increased tissue temperature or localized warmth [35].

International regulations impose strict requirements for the precise assessment and measurement of magnetic fields. For instance, the IEC/IEEE 63184 standard requires measurements at the closest possible distance to the system. However, performing measurements at a 0 mm distance is physically impossible due to the size of sensing probes and antennas. To satisfy this zero-contact requirement and ensure safety compliance, commercial systems typically employ extrapolation methods. For instance, designed for frequencies up to 100 kHz, Wavecontrol magnetic field probes, models WPF3, WPF6, and WPF8, utilize a sensor probe with a diameter of 6 cm [36].

Most commercially available sensors achieve precise magnetic field measurements without requiring probe rotation by utilizing three independents, orthogonally arranged coils. In this configuration, each axis is coupled to a rectifying diode that converts the high-frequency AC voltage–induced by the magnetic field–into a stable DC signal proportional to the square of the field strength. Capacitors then smooth this output to ensure a steady, readable value for the meter. Compared to Hall Effect sensors, these diode-based probes offer several advantages: a superior broadband response from kHz to GHz, the capability to capture rapid field pulses, and a passive sensing architecture that requires no power, thereby reducing interference [37].

## 3. Materials and Methods

This section details the design, fabrication, and interface circuitry of the inductive MEMS microsensor based on Faraday’s Law of Induction, specifically developed to detect and map time-varying magnetic fields within WPT systems. Fabricated using KLayout software [38] version 0.30.1 and CIDESI_TFS20 technology, the device features a multi-layered structure consisting of a silicon dioxide dielectric layer patterned via photolithography, a Ni/Cr metallic inductor, and a protective silicon nitride passivation layer. When penetrated by magnetic flux from a primary transmitter coil, the micro-fabricated inductor generates a proportional high-frequency Alternating Current (AC) voltage, typically ranging from 40 mV to 140 mV depending on proximity. To process this signal, an integrated interface circuit, which incorporates a rail-to-rail preamplifier, a peak detector, and an adder amplifier, converts the induced AC voltage into a stable direct current. This integration enables magnetic–to–electrical signal conversion, making the sensor suited for battery-operated portable devices, flux leakage detection, and electromagnetic compatibility assessments.

**Figure 2:**
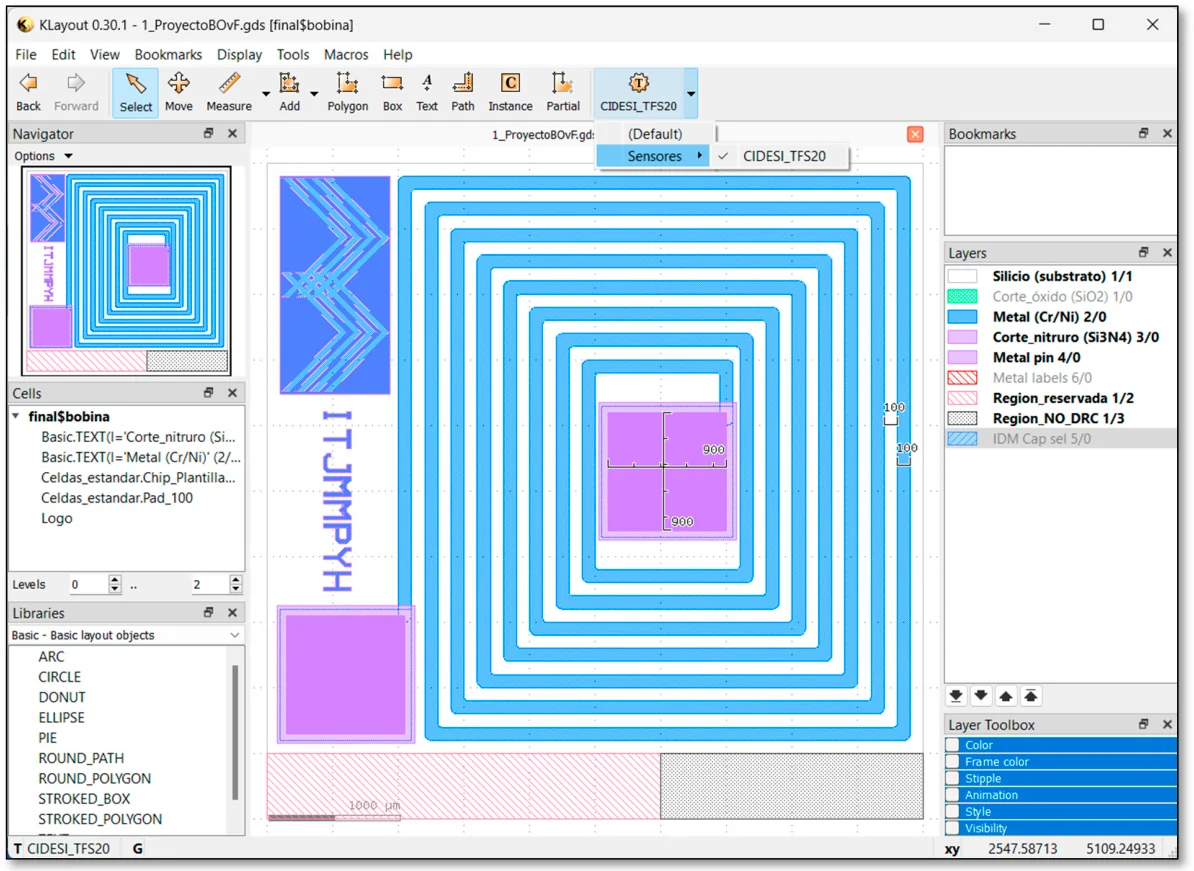
MEMS sensor layout designed in KLayout, compliant with CIDESI_TFS20 fabrication process constraints.

In this work, a MEMS sensor based on an inductor was designed and fabricated. The inductor was designed on the metal layer using Klayout software version 0.30.1 and under the constraints of the CIDESI_TFS20 fabrication technology, Querétaro, México. Figure 2 shows a screenshot of the KLayout interface, where the inductor is visible in the center, designed within a working area of 5 mm × 4.5 mm. The overall dimensions of the fabricated MEMS sensor are 5 mm × 5 mm ×1.15 mm. As illustrated in the layout, the Cr/Ni metal lines have a thickness and spacing of 0.1 mm, while the exposed wire-bonding pad (without the passivation layer) features a square area of 0.90 mm × 0.90 mm. Material layers are listed in the right-side menu. The hatched areas represent restricted spaces defined by the fabrication constraints. The sensor was fabricated in the laboratories of the Centro de Ingeniería y Desarrollo Industrial (CIDESI) in Querétaro, México. While the current prototype has a unit cost of approximately $3000 MXN, this cost is expected to decrease significantly with mass production.

**Figure 3:**
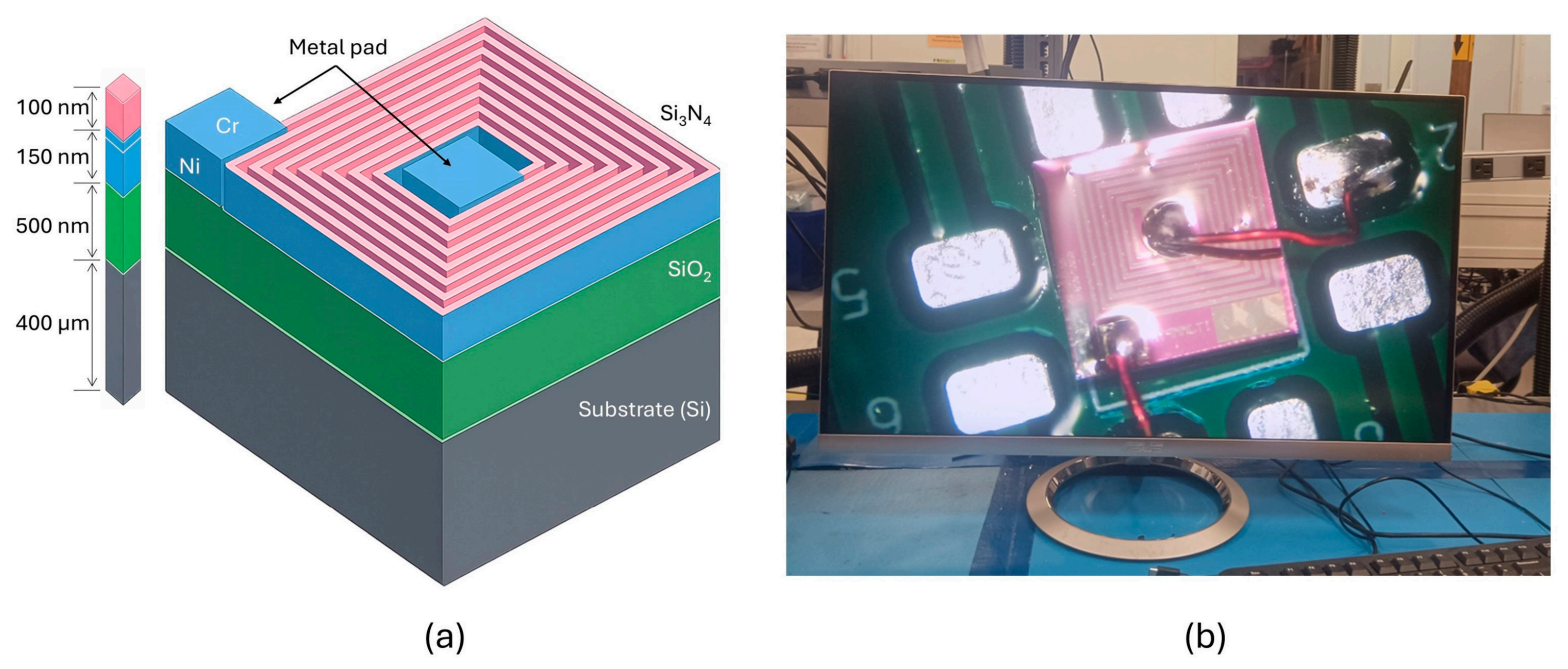
MEMS sensor: (**a**) material layers and (**b**) close-up of the wire bonds connected to the metal contact pads.

As shown in Figure 3a, a 500 nm thick silicon dioxide (SiO_2_) dielectric layer was deposited on a 400 μm thick, 25 mm^2^ silicon substrate using photolithography, followed by a 130 nm thick Ni and 20 nm thick Cr metallic layer. A 100 nm thick silicon nitride (Si_3_N_4_) layer was deposited as the top passivation layer to protect the metallic layer from moisture and contamination. Si_3_N_4_ is a high-hardness ceramic material capable of resisting mechanical wear. It has a low coefficient of thermal expansion, allowing it to withstand rapid temperature changes without fracturing. Furthermore, it is a dielectric and chemically stable material. As shown in Figure 3b, the MEMS sensor was attached to a PCB and electrically connected via wire bonding to the metal contact pads.

**Figure 4:**
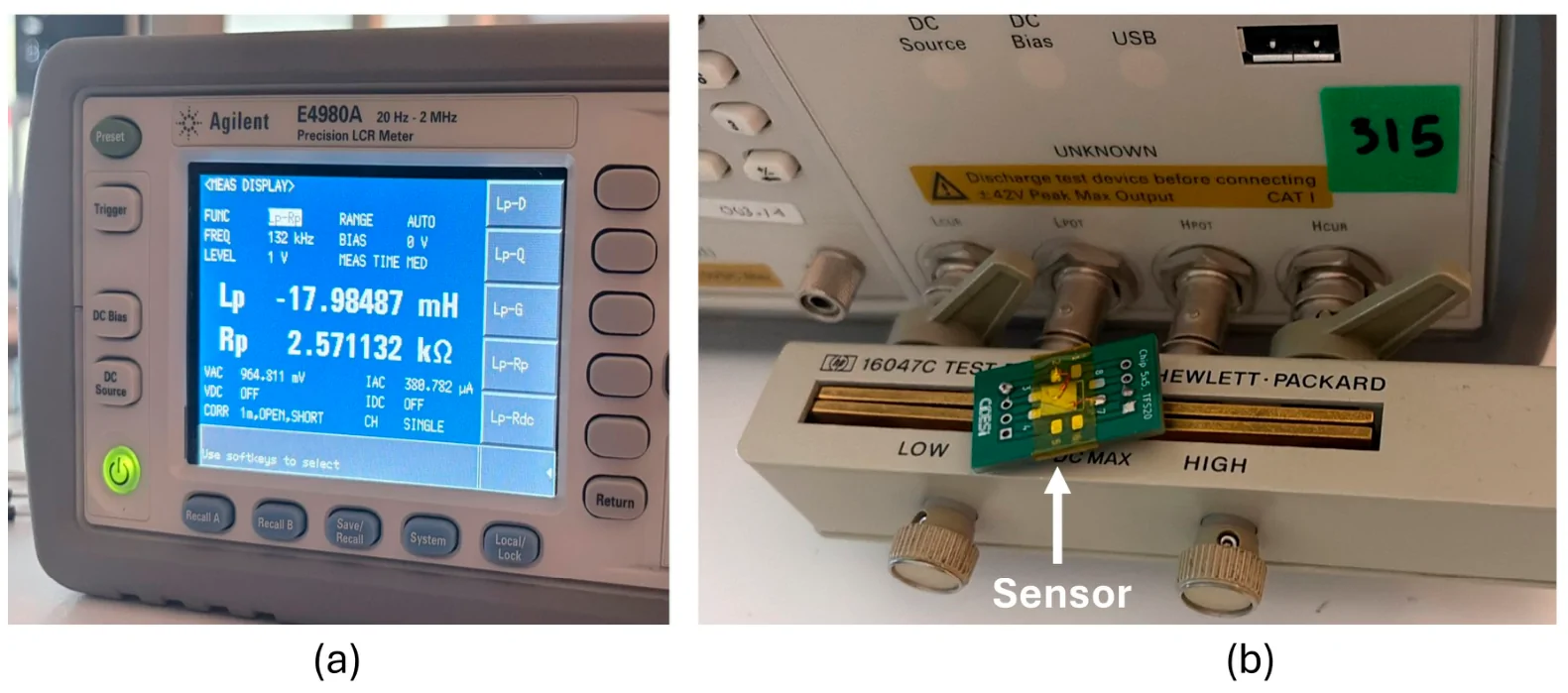
Characterization of the MEMS sensor: (**a**) Agilent E4980A LCR meter, (**b**) MEMS sensor connected to the Device Under Test (DUT) terminal.

The impedance of the MEMS sensor was characterized using an Agilent E4980A LCR Meter (Agilent Technologies, Inc., Santa Clara, CA, USA), as shown in Figure 4. Measurements were conducted across a frequency range from 20 Hz to 2 MHz, encompassing the 132.7 kHz operating frequency of the WPT Tx. A total of 48 discrete frequency points were sampled to ensure a comprehensive profile of the sensor’s performance. Measurements were performed using the L_p_ − R_p_, C_p_ − R_p_, L_s_ − Q, L_p_ − Q functions. L_p_ and C_p_ represent the parallel-equivalent inductance and capacitance, while R_p_ denotes the equivalent parallel resistance. L_s_ refers to the series-equivalent inductance, and Q is the quality factor or the inverse of the dissipation factor [39].

**Figure 5:**
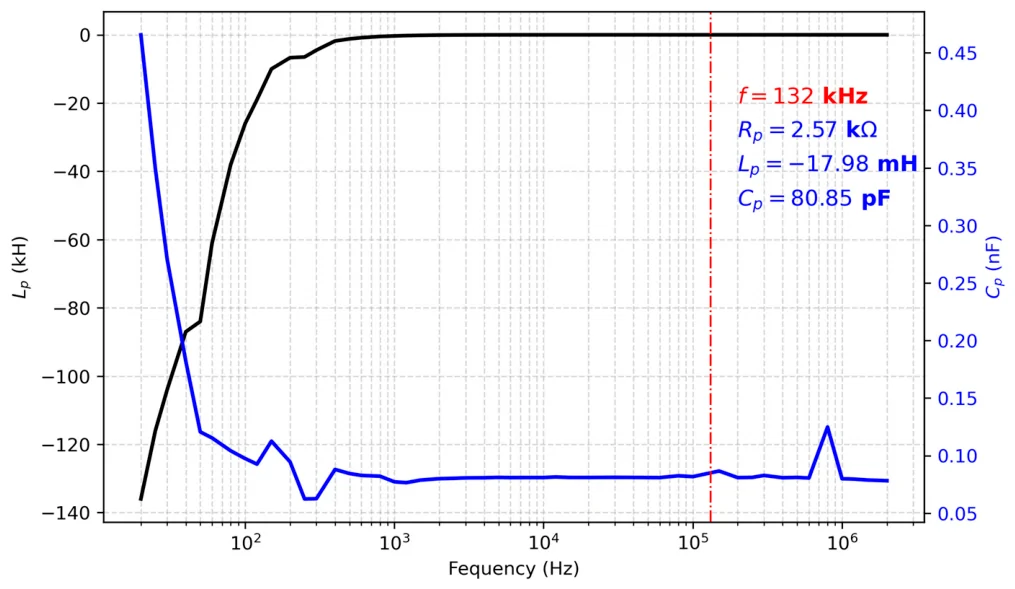
MEMS sensor impedance measurements.

As shown in Figure 5, the capacitance exhibits a sharp decline from 0.45 nF to below 0.1 nF within the 20 Hz to 1 kHz range, stabilizing thereafter up to 2 MHz. At the target frequency of 132 kHz, the sensor presents a parallel resistance (R_p_) of 2.57 kΩ and a parallel capacitance (C_p_) of 80.85 pF. The high resistive profile of the sensor is a direct result of the nickel thin-layer dimensions. Utilizing a layer thickness of 130 nm and a width of 100 µm, the resistance for a 20 µm segment is 0.64 Ω. When extrapolated over the inductor’s total length of 4378 µm, the theoretical resistance reaches 2.8 kΩ, effectively validating the experimental measurements.

The negative inductance value of L_p_ = −17.98 mH shown in Figure 5 is not a non-physical effect of the sensor. Instead, it is a characteristic result of the Agilent E4980A LCR meter’s equivalent circuit model when capacitive reactance dominates the device’s response X_C_ > X_L_. Despite the sensor featuring a Ni/Cr metallic microcoil, the thin-layer inductive effect is dominated by the device’s capacitance at 132 kHz. The nickel thin layer results in a significant internal resistance; this, combined with a low-quality factor (Q) magnitude, indicates that energy dissipation is high relative to the stored reactive energy. Additionally, the silicon nitride passivation layer acts as a diamagnetic insulator, contributing to the capacitive behavior of the sensor.

**Figure 6:**
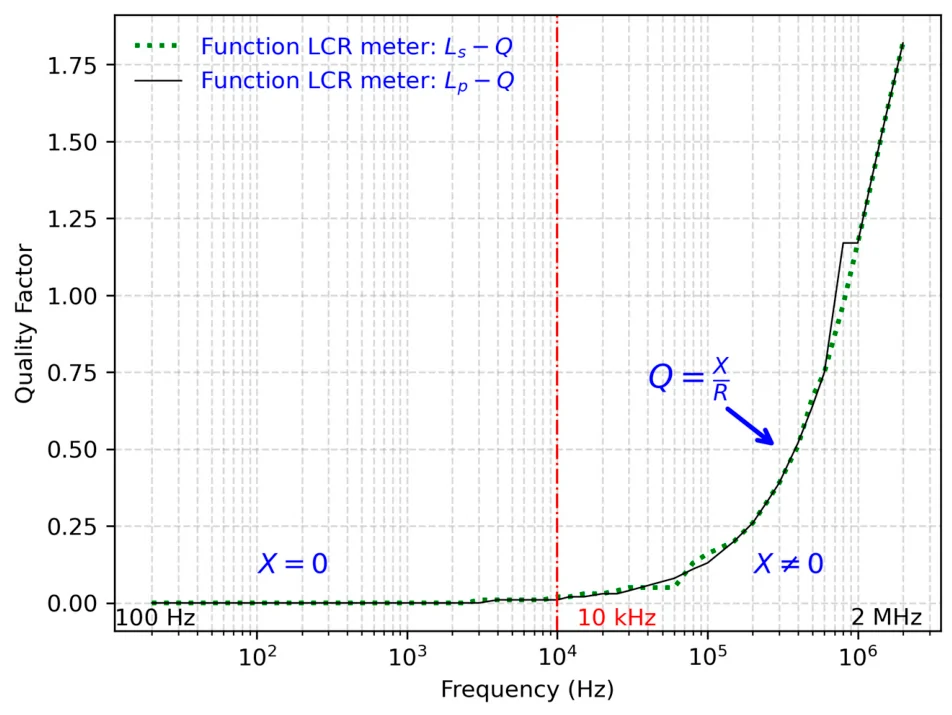
Quality factor versus frequency.

The frequency response of the quality factor is presented in Figure 6. Below 10 kHz, Q is negligible, signifying a purely resistive response. Above this frequency, the quality factor rises to 1.82 at 2MHz. Such a low Q magnitude indicates a high rate of energy dissipation relative to the reactive energy stored, consistent with the sensor’s characterization at these operating frequencies. Stored energy in precision sensors often leads to oscillations that degrade signal stability. Sensors with low energy storage are preferable as they provide an immediate response to changes.

Beyond precision, a high-quality sensor must exhibit minimal hysteresis to ensure it returns accurately to its zero−point following magnetic exposure. This reliability is essential to the requirements for next-generation magnetic sensing, combining signal stability and a near−instantaneous response with practical constraints such as small size, low power consumption, and low cost. Additionally, these sensors are increasingly designed for resilience in aggressive media and simplified operation, allowing them to be operated by non-skilled personnel [40].

Regarding the electrical characterization presented in Figure 5 and Figure 6, the measurements were obtained directly from the high−precision Agilent E4980A LCR meter. Under consistent laboratory conditions, multiple frequency sweeps from 20 Hz to 2 MHz revealed no perceptible discrepancies in the sensor’s impedance or quality factor.

**Figure 7:**
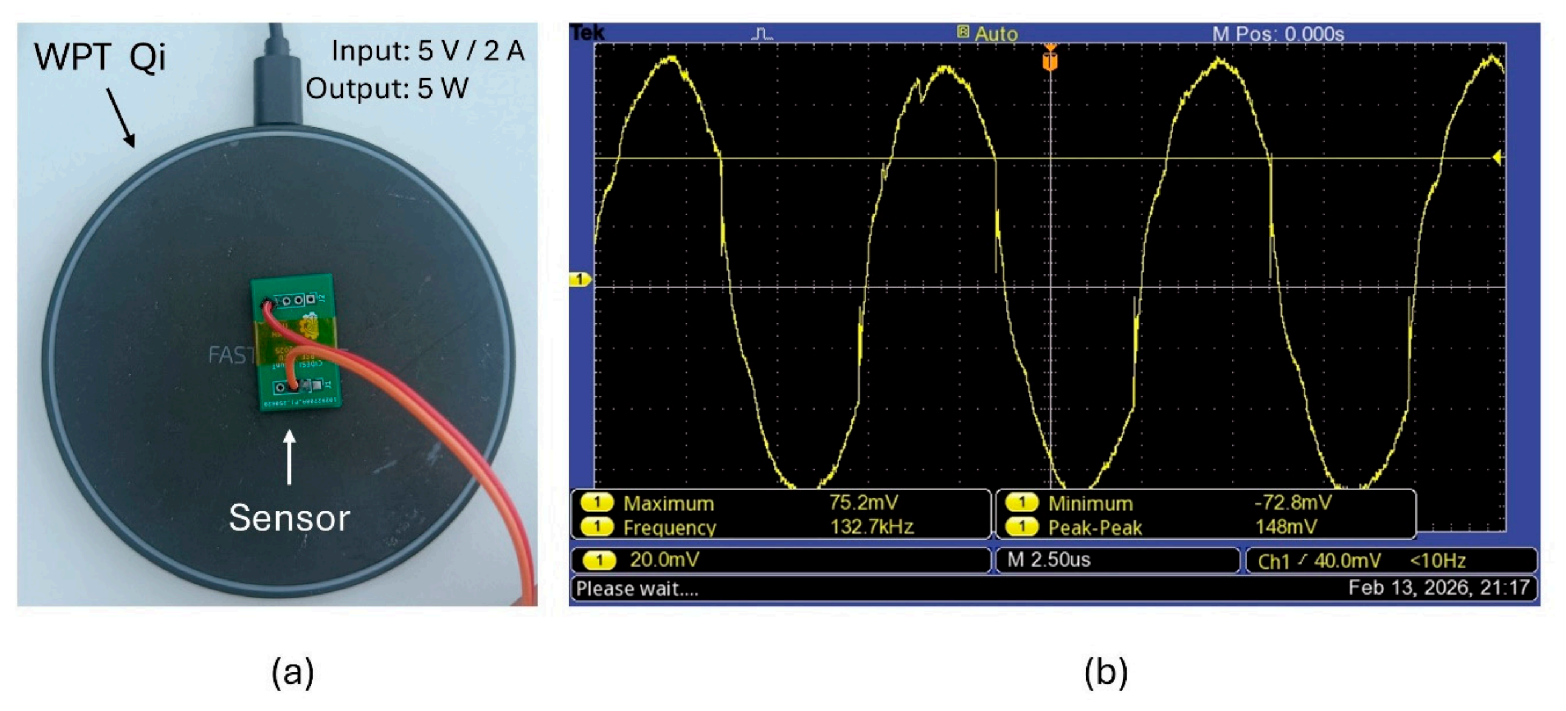
Experimental setup and signal acquisition: (**a**) MEMS sensor positioned in proximity to a Qi-standard wireless charger, and (**b**) corresponding signal detected by the sensor.

Figure 7 shows the sensor signal under zero−contact conditions with a commercially available Qi−standard wireless charger. The device specifications include an input of 5 V and 2 A, an output of 5 W, and a diameter of 98 mm. Measurements were performed using a Tektronix TBS1102B-EDU oscilloscope (Tektronix, Inc., Shanghai, China). The sensor signal shown in Figure 7b has a frequency of 132.7 kHz and a 148 mV peak−to−peak voltage.

**Figure 8:**
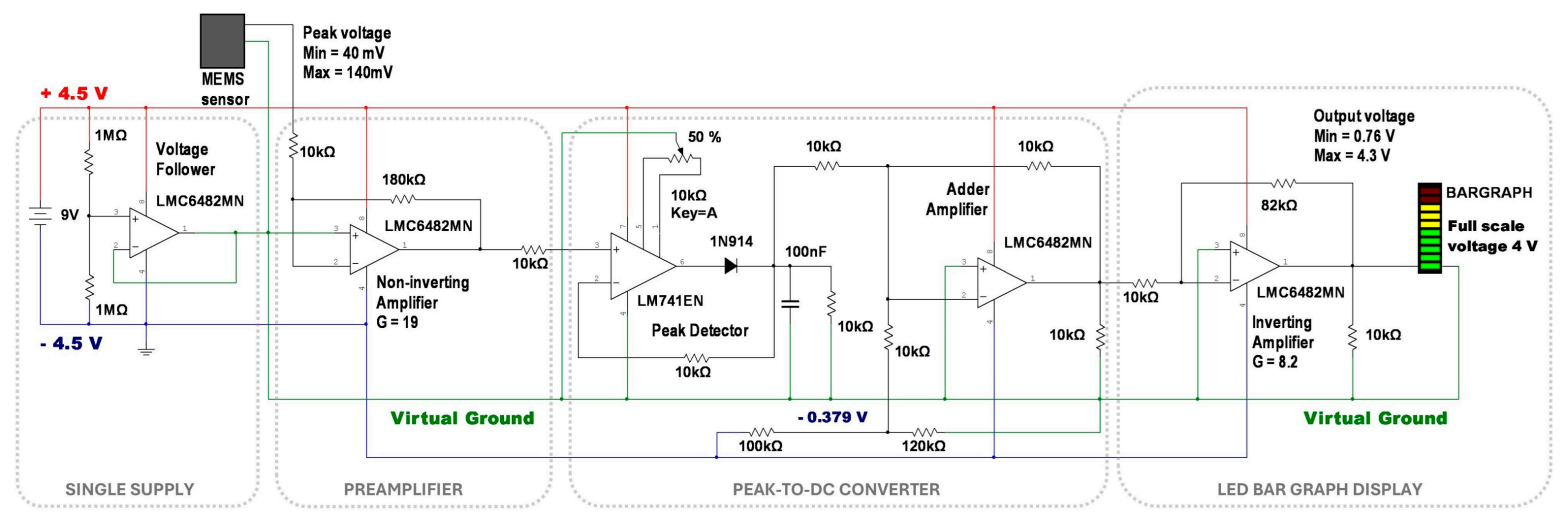
MEMS sensor interface circuit blocks: Single supply, Preamplifier, Peak−to−DC converter, and LED bar graph display.

Figure 8 illustrates the MEMS sensor interface circuit designed for magnetic field strength detection using a single 9 V supply and a virtual ground. As the distance from the WPT transmitter decreases from 30 mm to 0 mm, the sensor’s peak AC output scales from 40 mV to 140 mV. This signal is initially elevated by an LMC6482 rail-to-rail operational amplifier with a gain of 19. The rail–to–rail architecture is ideal for this low-voltage application, as it minimizes voltage drops across the output transistors to maintain a high signal-to-noise ratio and a wide dynamic range. The amplified AC signal is then processed by an LM741-based peak detector to produce a stable DC representation. To refine the output, a summing amplifier applies a −0.379 V offset, shifting the signal floor to suppress low-level noise. Finally, an inverting stage scales the DC signal to a range of 0.76 V to 4.3 V. This output range is optimized for driving an LED bar graph or for interfacing with an analog–to–digital converter, where the voltage can be parameterized to represent magnetic field strength in a microcontroller environment. It is important to note that the use of a single-supply configuration allows the device to be powered by a battery, thereby facilitating its design as a portable instrument. The proposed circuit was simulated in Multisim and validated on a solderless prototyping board. Future work includes the design of a custom printed circuit board and a protective plastic enclosure.

## 4. Results

This section presents a comprehensive evaluation of the proposed compact MEMS-based magnetic field induction sensor. The findings are organized into three sequential subsections. Section 4.1 outlines the simulation results using the Python Magpylib library version 5.2.3, which evaluates the induced voltage of the concentric MEMS sensor under vertical displacement and under lateral displacement at a constant gap. Section 4.2 analyzes the relationship between transmission gaps, the processing of raw AC waveforms into DC signals for real-time diagnostic output, and spatial magnetic field mapping to identify leakage “hot spots” critical for effective thermal management. Section 4.3 provides physical validation using infrared thermograms to observe the correlation between the temperature and the MEMS inductive sensor readings.

### 4.1. Inductive Coupling Simulation

The simulations of the inductive coupling between the transmitter coil and the sensor were performed using the Magpylib Python library version 5.2.3, [41]. While Magpylib is primarily designed to compute static magnetic fields and lacks a direct function to calculate inductance, the coupling can be obtained by applying the fundamental equation for the total magnetic flux, $\Phi_{\mathrm{total}}$, through a surface. In (1), $I_{1}$ represents the current flowing through the transmitter coil, $B_{1}$ is the magnetic field generated by the transmitter coil, and $S_{i}$ is the area enclosed by each of the n concentric turns of the sensor.

(1) $$M=\frac{\Phi_{\mathrm{total}}}{I_{1}}=\frac{1}{I_{1}}\sum_{i=1}^{n}\iint_{S_{i}}B_{1}\cdot dS_{i}$$

To calculate the electromagnetic coupling, a dense 2D grid of points is generated within the area of each sensor turn. Subsequently, the magnetic field produced by the transmitter coil is evaluated at each grid point, incorporating the spatial position of the sensor. The differential area is determined through a numerical approximation, and a summation is applied to approximate the surface integral of the magnetic flux. From this flux, the mutual inductance is calculated, and finally, the induced voltage (2) is obtained at an operating frequency of 132.5 kHz. The induced voltage is multiplied by the preamplifier gain (G = 19) of the MEMS sensor interface circuit to approximate the amplified output voltage shown on the right *y*-axis.

(2) $$V=2\pi \cdot f\cdot M\cdot I_{1}$$

**Figure 9:**
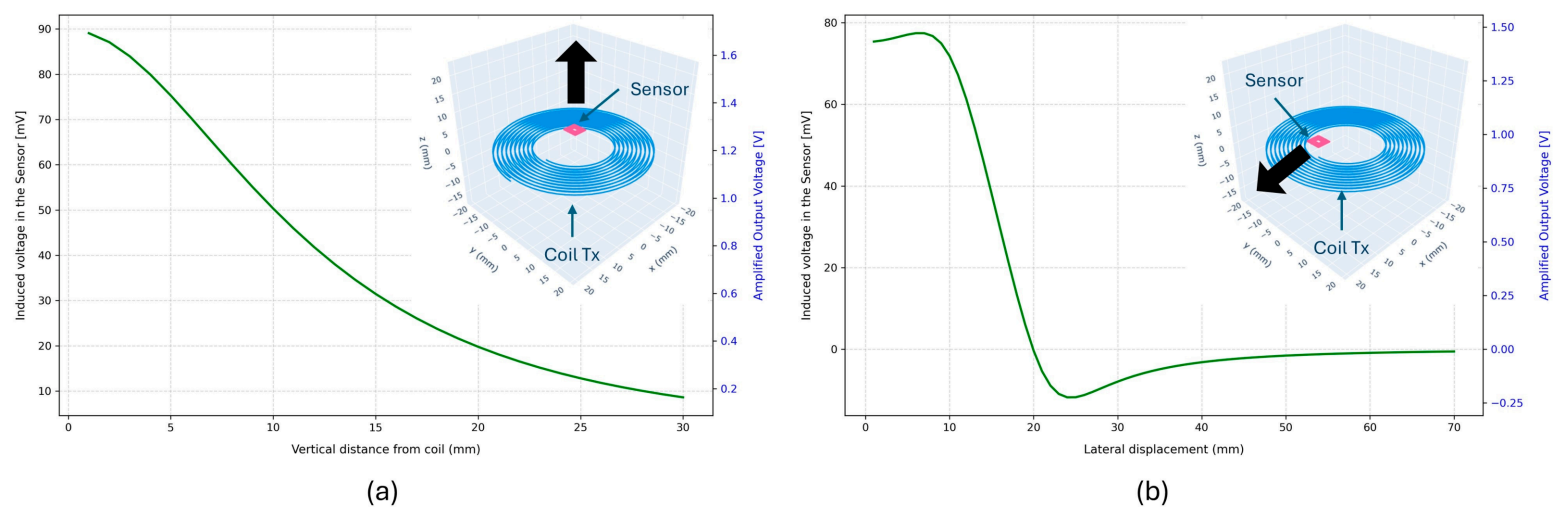
Simulation of the induced voltage in the sensor: (**a**) vertical displacement under concentric alignment, and (**b**) lateral displacement at a constant vertical gap of 5 mm.

Figure 9a presents the simulated induced voltage and the corresponding amplified output voltage as a function of the vertical distance, swept from 1 mm to 30 mm under concentric alignment. The transmitter coil—configured with an inner radius of 10.25 mm, an outer radius of 21.5 mm, and 20 turns distributed across 2 layers with a 1.0 mm wire diameter—was excited with a current of 2.0 A. In response, the MEMS sensor, featuring 8 concentric turns with outer dimensions of 3.9 mm × 4.3 mm, a metallic conductor diameter of 0.1 mm, and a 0.1 mm inter-turn spacing, exhibits a strong inductive coupling at close proximity. At a minimum vertical gap of 1 mm, the theoretical induced voltage reaches its peak of approximately 89.1 mV, corresponding to an amplified output voltage of about 1.65 V. As the vertical separation increases, the induced voltage undergoes a non-linear, exponential-like decay due to the rapid attenuation of the magnetic field. At the maximum distance of 30 mm, the induced voltage decreases to approximately 8.6 mV, yielding an amplified output of approximately 0.16 V.

**Figure 10:**
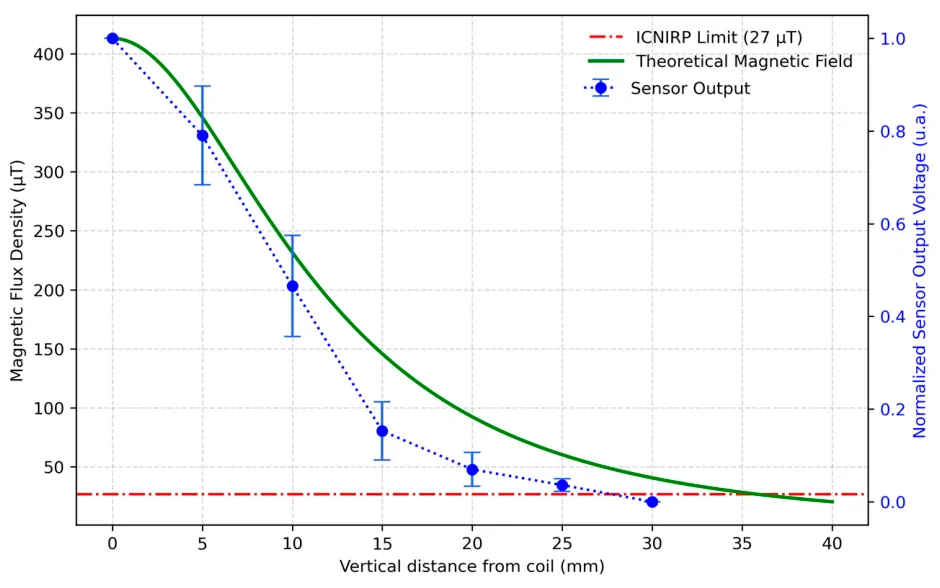
Decay of the magnetic field as a function of vertical gap.

Figure 9b shows the sensor’s response under lateral displacement, swept along the positive X-axis from 1 mm to 70 mm at a constant vertical gap of 5 mm. Initially, at small displacements (X<10 mm), the induced voltage remains highly stable, exhibiting a peak of approximately 77.5 mV (~1.42 V amplified) around 7 mm. This slight increase occurs as the compact MEMS sensor transitions directly over the high-flux-density winding region of the transmitter coil. Beyond 10 mm, the induced voltage drops sharply, crossing the zero-voltage threshold at exactly 20 mm. For lateral displacements exceeding 20 mm, the induced voltage becomes negative. This phase reversal is a direct physical consequence of the closed-loop nature of magnetic field lines. Because the transmitter coil’s physical outer radius is 21.5 mm, moving the sensor beyond 20 mm positions it within the magnetic field’s return path. As the lateral displacement extends further toward 70 mm, the return-path field strength gradually dissipates, causing the sensor’s output to asymptotically approach zero. Since the MEMS sensor interface circuit has a noise floor (~0.075 V estimated at a vertical separation of 0 mm) higher than the voltage amplitude in the phase-reversal region, the signal remains masked by the noise. This is not detrimental to the sensor’s application, whose objective is to detect induction peaks in regions that do not exhibit the ideal behavior illustrated in Figure 10.

### 4.2. Experimental Characterization and Magnetic Field Mapping

Figure 10 illustrates the theoretical decay of the magnetic field for an A11 standard coil alongside the normalized output voltage of the proposed sensor as a function of vertical gap. While the A11 coil reaches the defined safety limit at 40 mm, the sensor output voltage becomes negligible beyond 30 mm. The theoretical values of the magnetic field were modeled using the Python Magpylib library [40], which utilizes analytical equations based on the Biot-Savart Law for current sources with circular symmetry. In (3), $\mu_{0}$ is the magnetic permeability of vacuum, I is the electric current, R is the loop radius, and z is the vertical distance above the loop.

(3) $$B_{\mathrm{total}}\left(z\right)=\sum_{i=1}^{N}\frac{\mu_{0}\cdot I\cdot R_{i}^{2}}{2{\left(R_{i}^{2}+z^{2}\right)}^{3/2}}$$

The industry−standard A11 design is the benchmark for 5 V, 5 W wireless charging. It utilizes a 10-turn, single-layer spiral coil with an outer radius of 22.0 mm and an inner radius of 10.25 mm, typically used in single-coil charging pads. In consumer electronics, most WPT systems operate within a 0−5 mm separation range, as magnetic field strength and coupling efficiency diminish rapidly with increased distance [42,43]. In practical applications, every additional 1 mm of distance can result in a 10% to 20% loss in charging efficiency. Once the cumulative gap inclusive of case thickness, air gaps, and alignment offsets exceeds the 5 mm threshold, energy transfer becomes unsustainable.

In Figure 10, the experimental data show a more pronounced decrease than the theoretical model at greater distances. This is because the magnetic induction in the Ni/Cr thin-film coils decays significantly, becoming insufficient to overcome the processing threshold of the interface circuit. This result implies that the sensor’s functional limit is less than 30 mm. Beyond these functional boundaries, user safety remains a critical design constraint. To balance this performance with user safety, these systems must strictly adhere to ICNIRP guidelines and limit the magnetic field to 27 µT to mitigate potential biological risks. Figure 10 also shows that the sensor’s operational range spans from 0 to 30 mm of vertical distance from the transmitter coil. Within this range, the magnetic flux density varies from approximately $B_{max}\approx 400\;\mu T$ at zero separation to $B_{min}\approx 40\;\mu T$ at 30 mm, while the induced voltage changes from $V_{max}\approx 89\;mV$ to $V_{min}\approx 9\;mV$, yielding an approximate sensor sensitivity of S=∆V/∆B= 0.216 mV/μT.

**Figure 11:**
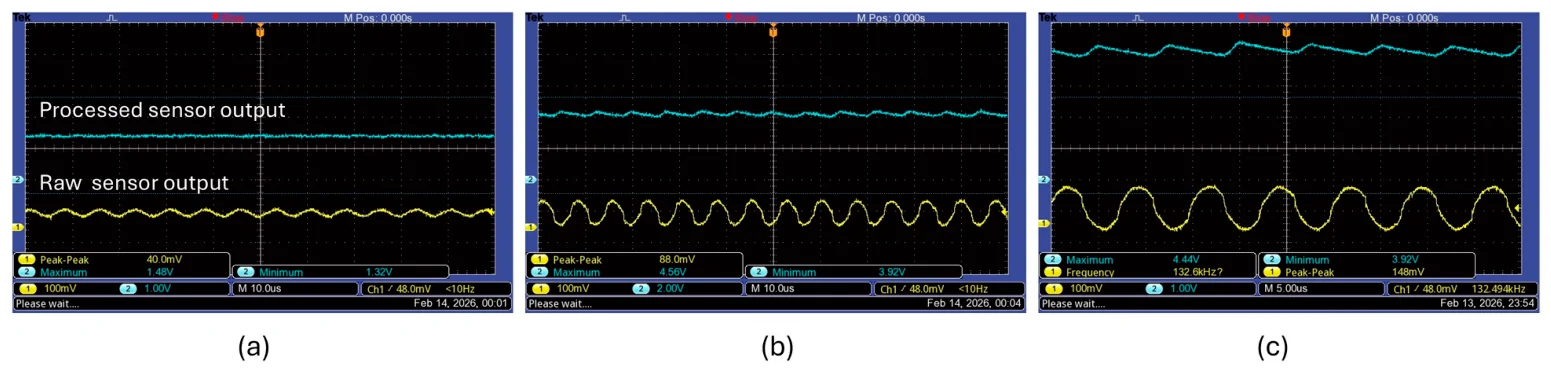
Comparison of raw and processed sensor signals as a function of magnetic field intensity for three sensor-to-WPT transmitter gaps: (**a**) far, (**b**) intermediate, and (**c**) close.

Figure 11 depicts the comparison of raw and processed sensor signals as a function of magnetic field intensity for three sensor−to−WPT transmitter distances. Waveforms were acquired using a Tektronix TBS1102B-EDU oscilloscope. The raw sensor output is an AC signal whose peak voltage increases with magnetic field intensity. As the gap between the sensor and the transmitter coil decreases, the peak voltage rises accordingly. Following signal processing, the output is converted to a DC signal with a small ripple. At a minimal separation, the processed DC signal fluctuates between a minimum of 3.92 V and a maximum of 4.44 V due to this ripple. This increased ripple under close-range conditions is attributed to the larger induced peak voltage, which increases the absolute discharge rate of the RC filter (RC=1 ms), and the charging current limitations of the operational amplifier when driving the 100 nF capacitor at 132.5 kHz. In practice, this level of ripple voltage is negligible in practice and does not affect the performance or stability of the subsequent LED bar−graph display. It should be noted that the final DC output voltage of the processed signal is governed by the gain and compensation parameters of the interface circuitry.

**Figure 12:**
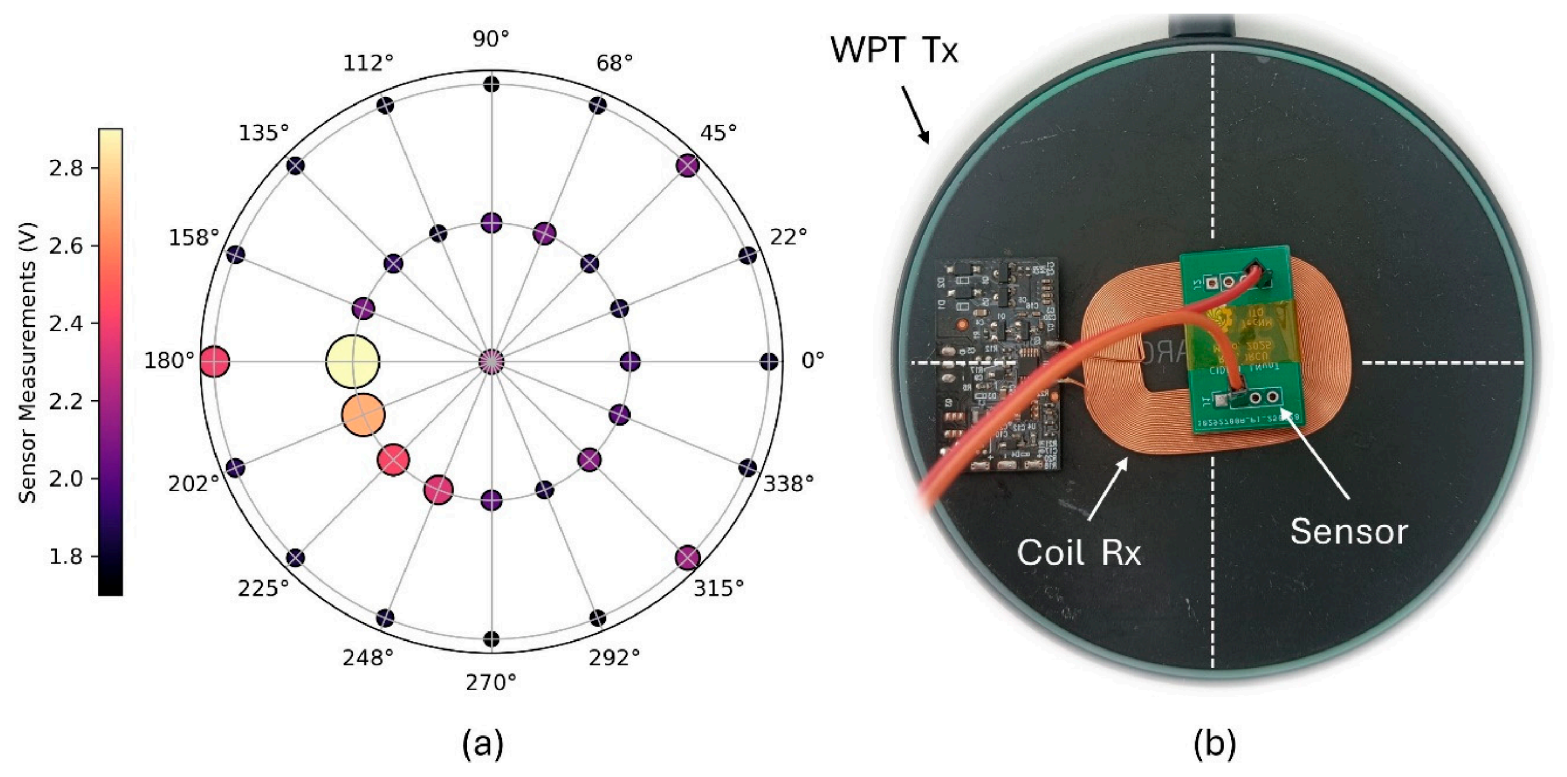
Spatial magnetic field characterization: (**a**) Polar plot of sensor voltage measurements across the charging surface; (**b**) Experimental setup showing the WPT transmitter, receiver coil, and sensor.

Figure 12 illustrates the characterization of the magnetic field distribution using the proposed sensor. The experimental setup, depicted in Figure 12b, consists of a Rx coil centered over a WPT Tx, with the sensor positioned directly above the Rx coil. The resulting spatial measurements are mapped in the polar plot shown in Figure 12a, where both the diameter and color of the data points represent the sensor’s output voltage.

The results indicate the peak magnetic field intensity is concentrated near the interface between the receiver coil and its associated circuitry. These localized hot spots indicate regions of significant magnetic leakage. Identifying these fields is crucial for Electromagnetic Compatibility (EMC) and thermal management. By mapping these leakage zones, designers can optimize the placement of internal metallic components—such as shielding, structural frames, or battery casings—to prevent unintended parasitic induction. This mitigation is essential to avoid localized heating in conductive parts of the charging device that are not part of the WPT system.

It should be noted that the total magnetic field perceived by the sensor is a superposition of the primary field from the transmitter coil and the scattered field generated by the sensor’s own metallic structure. Although these local field interactions—including nonzero components in the XY plane—are factors to consider when evaluating precision against ideal analytical models, it does not compromise the primary focus of this work. The sensor is specifically designed for the identification of magnetic leakage and localized hot spots, as demonstrated by the spatial characterization in Figure 12; the sensor effectively identifies regions of high magnetic intensity, carrying out its role as a practical tool for mapping leakage hot spots, ensuring EMC, and mitigating thermal risks in WPT systems.

### 4.3. Thermal Validation

**Figure 13:**
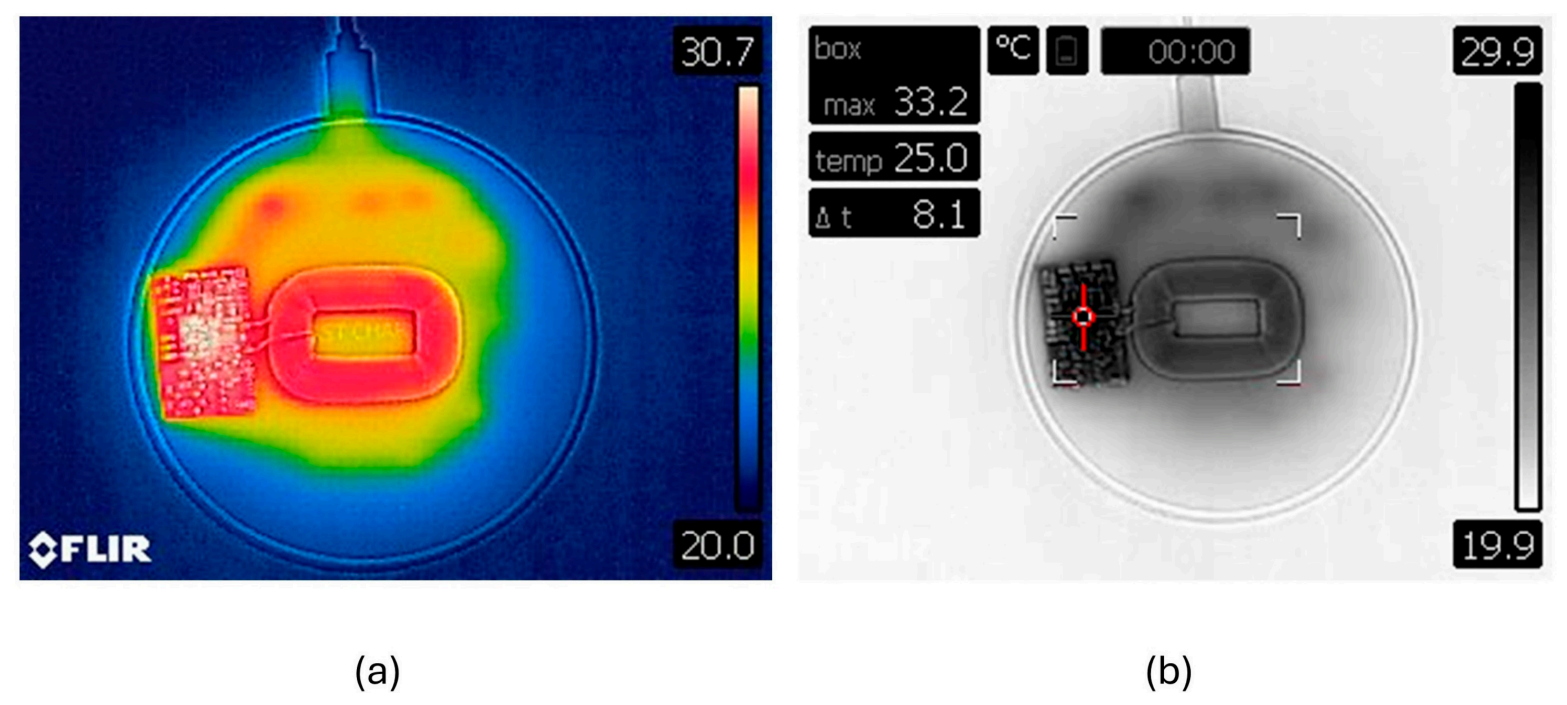
Thermal characterization of the receiver: (**a**) Distribution of radiated heat from the Rx onto the Tx pad; (**b**) Measurement of the peak temperature at the primary hot spots.

As shown in Figure 13a, the Rx was positioned relative to the Tx using the same configuration established for the spatial magnetic field characterization in Figure 12a. Although circuit operation is the main driver of the temperature increase, direct physical contact with the transmitter pad accelerated the time to reach 30 °C by 5 to 10 s compared to separations of 5, 10, and 15 mm. This behavior occurs because increased magnetic induction enhances inductive heating within the receiver circuitry.

A FLIR E50 handheld thermal camera, characterized by a 240 × 180-pixel resolution and a thermal sensitivity of <0.07 °C, was used to observe the correlation between the temperature and the MEMS inductive sensor readings. Imaging was performed using the Thermal MSX mode, which enhances infrared captures by overlaying visible-spectrum outlines and edges onto the thermal image in real-time to provide greater structural detail. As shown in Figure 13a, the receiver circuit and coil exhibit temperatures higher than the environment, with heat radiating toward the transmitter pad. Figure 13b identifies the circuit center as the primary thermal hot spot at 33.1 °C, corresponding to an 8.1 °C temperature rise above the ambient baseline of 25 °C.

**Figure 14:**
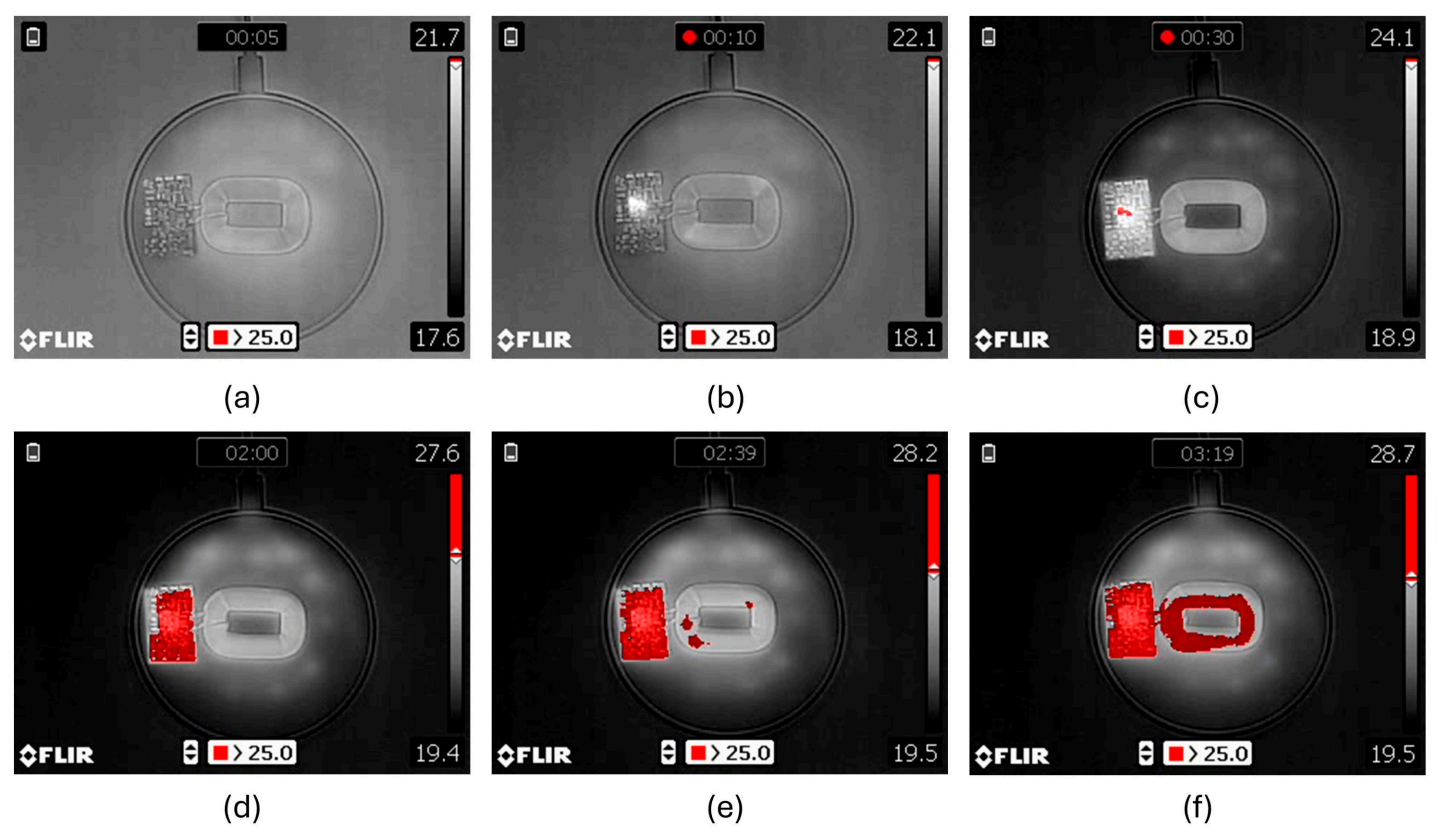
Sequential infrared thermograms illustrating the heat dissipation profile at specific time intervals: (**a**) 5 s, (**b**) 10 s, (**c**) 30 s, (**d**) 2 min, (**e**) 2 min 39 s, and (**f**) 3 min 19 s. The red isotherms indicate areas exceeding the 25.0 °C threshold.

The infrared thermograms in Figure 14 illustrate the heat dissipation profile at discrete time intervals. Upon activating the WPT Tx, no significant thermal deviation from the ambient room temperature was detected within the first 5 s. After 10 s, a localized temperature rise began at the circuit’s center, as depicted in Figure 14b. To clearly identify critical thermal zones, a threshold of 25.0 °C was established. After 2 min, the receiver’s PCB temperature exceeded this 25.0 °C threshold. At 2 min and 39 s, red isotherms indicate the emergence of a hot spot within the Rx coil. Finally, at 3 min and 19 s, the majority of the coil surface and PCB exceeded the established temperature limit.

## 5. Discussion

The proposed MEMS device functions as a specialized induction microsensor based on Faraday’s Law [2], utilizing a micro-fabricated Ni/Cr metallic inductor to detect time-varying magnetic fields in Wireless Power Transfer (WPT) systems. The integrated interface circuit, comprising a rail-to-rail preamplifier and a peak detector, effectively converts high-frequency AC signals into a stable DC representation proportional to the field intensity. Notably, the sensor’s low-quality factor (Q) at 132 kHz is a key design advantage, as it minimizes unwanted oscillations and provides a near-instantaneous response to rapid field variations. This architecture ensures reliable signal capture while maintaining compliance with international safety guidelines such as ICNIRP [35].

Experimental results confirm that the sensor output closely follows the theoretical magnetic field decay of an industry-standard A11 coil, although functional detection is effectively limited to a 30 mm separation distance. Beyond this threshold, the induced signal becomes insufficient to overcome the processing floor of the interface circuitry. The device successfully identifies localized “hot spots” near coil-circuitry interfaces, providing a practical framework for validating theoretical models in consumer-grade systems [12]. By mapping these leakage zones, designers can optimize the placement of internal metallic components to prevent parasitic induction and hazardous thermal incidents, which were shown to reach temperatures over 50 °C in unmonitored scenarios.

The practical utility of the proposed sensor lies in its ability to provide localized and quantitative information that is not available from a simple visual or thermal inspection of the charger. Although the general decrease in magnetic field with distance and the heating of conductive foreign objects are well-known effects in WPT systems, the proposed sensor enables these phenomena to be spatially resolved and electrically quantified. In the experimental tests, the sensor output followed the expected magnetic-field decay of the transmitter coil and became negligible beyond approximately 30 mm, defining a practical diagnostic range for near−field WPT evaluation. Within this range, the measured voltage response can be used to identify regions where magnetic coupling is stronger, weaker, or locally distorted by the geometry of the receiver coil, nearby circuitry, or conductive objects. This capability is particularly useful during charger design and validation, where engineers need to locate leakage hot spots, evaluate coil alignment, compare shielding strategies, and identify regions where parasitic induction may cause unwanted heating.

Based on the spatial mapping and thermal validation results, the proposed sensor can be applied as a diagnostic tool in several practical scenarios. In product development, it can support the optimization of transmitter and receiver coil placement by revealing the regions of maximum magnetic coupling and leakage. In quality-control testing, it can provide a rapid method to compare the magnetic-field distribution of different charger units and detect abnormal field patterns caused by assembly tolerances or defective components. In electromagnetic compatibility evaluation, it can help identify leakage regions that may interfere with nearby electronics or conductive structures. In foreign-object and thermal-risk analysis, the sensor can be used together with infrared thermography to correlate magnetic hot spots with temperature rise, allowing early identification of potentially hazardous operating conditions. Therefore, the value of the proposed device is not only its absolute magnetic sensitivity, but its compact size, simple readout circuit, and ability to perform localized WPT field diagnostics under realistic operating conditions.

Future research will focus on evaluating the sensor across diverse power levels, operating frequencies, and complex coil geometries to enhance its diagnostic capabilities for next-generation, high-capacity WPT systems. While testing at higher induction levels is expected to improve the signal-to-noise ratio, these advancements must be carefully balanced against potential increases in circuit complexity and power consumption [30,31,32]. Additionally, developing a sensor array inspired by Hall-effect configurations [23,27,40] will enable real-time magnetic field mapping, thereby dispensing with manual probe positioning and facilitating fully automated diagnostic instruments.

## 6. Conclusions

In this study, a MEMS-based induction sensor was successfully fabricated using photolithography, incorporating a metallic inductor layer composed of 130 nm Ni and 20 nm Cr. To ensure long-term reliability, a silicon nitride passivation layer was deposited to protect the metallic structures from moisture ingress and environmental degradation.

The MEMS sensor, featuring a compact layout of 5 mm × 5 mm, was integrated onto a PCB and interfaced with a dedicated signal conditioning stage. This stage employs rail-to-rail operational amplifiers in a single-supply configuration and a peak-detection circuit to process the induced low-amplitude voltage. The resulting output is scaled to a dynamic range compatible with either an LED bar-graph display or a microcontroller’s analog-to-digital converter.

Experimental characterization confirms that the sensor output voltage closely follows the theoretical magnetic field decay as the vertical separation increases, becoming negligible beyond 30 mm while the maximum output occurs at zero separation. Furthermore, the sensor effectively maps spatial magnetic field distributions, identifying critical leakage hot spots near coil-circuitry interfaces. By providing precise spatial diagnostics of magnetic leakage, the proposed sensor represents a valuable tool for improving EMC compliance and thermal safety, ultimately enabling the mitigation of parasitic induction and preventing hazardous localized heating in wireless charging devices.

## Figures and Tables

**Table 1:** Comparison of common magnetic sensors.

Sensor	Resolution (T)	Advantage	Disadvantage	Common Application
SQUIDs (Superconducting Quantum Interference Devices) [13]	10^−15^	Operating at cryogenic temperatures to suppress thermal noise, SQUIDs offer unsurpassed sensitivity in magnetic field detection.	The practical deployment of SQUIDs is constrained by their bulky size, electronic complexity, and the high costs associated with mandatory cryogenic cooling.	These sensors are used in medical imaging and nondestructive evaluation in materials science.
MO (magneto-optical) [14,15]	10^−12^	Magneto-optical sensors utilize light for signal transmission, providing immunity to electromagnetic noise and a non-conductive property ideal for high-voltage power lines.	Thermal sensitivity in magneto-optical materials can cause signal drift, necessitating complex optics and stable lasers to maintain accuracy	These devices are used for monitoring current in high-voltage lines and detecting subsurface cracks in aircraft fuselages.
FGM (FluxGate Magnetometer) [16]	10^−12^	They precisely measure the strength and direction of weak, static, and low-frequency magnetic fields, offering exceptional stability for long-term monitoring.	Because they require a physical magnetic core and wire coils, they are significantly bulkier than MEMS or Hall effect sensors.	These sensors are used in geophysics observation, mineral and oil exploration, and archeology.
MI (Magneto Impedance) [17,18]	10^−12^	Suitable for mass production and integration, these sensors feature high sensitivity, linear response, and low noise levels in unshielded environments.	The sensors are susceptible to high-frequency EMI and possess high directional sensitivity; any angular misalignment relative to the field leads to substantial accuracy loss.	These sensors provide real-time measurements of human biomagnetic signals and variations in the Earth’s geomagnetic field.
GMR (Giant MagnetoResistance) [19]	10^−9^	They offer high sensitivity, a wide frequency range, small size, and low power consumption.	Their accuracy is often limited by nonlinearity, hysteresis, offset, and temperature dependency; furthermore, unipolar variants are restricted in AC measurement applications	This type of sensor is utilized in hard drive read heads, automotive Anti-Lock Braking Systems (ABS), speed sensors, and industrial current sensors
TMR (Tunneling Magneto Resistance) [20]	10^−12^	TMR sensors outperform GMR sensors in sensitivity and power efficiency. Due to their higher resistance, they consume less power than GMR sensors at the same operating voltage.	They suffer from higher noise. Sensors are more expensive and difficult to fabricate. However, TMR sensors offer the advantage of being fabricable on flexible organic substrates	Due to the temperature sensitivity of TMR device resistance in the anti-parallel state, these sensors are ideal for temperature monitoring and integrated circuit overheat protection.
AMR (Anisotropic MagnetoResistance) [21,22]	10^−6^	They have lower sensitivity than GMR and TMR sensors and have better Signal-to-Noise Ratio (SNR) at low frequencies.	Permalloy, an iron-nickel alloy, is the most widely used material for AMR sensors; its specific composition is critical for achieving negligible magnetostriction	High paramagnetic susceptibility makes oxygen highly selective for AMR sensors, enabling detection across the full 0–100% range
HES (Hall Effect Sensor) [23]	10^−6^	Widely available from commercial suppliers, these compact, low-cost, and low-power sensors integrate easily into systems to provide real-time magnetic field measurements	These sensors require high-gain amplification that introduces electronic noise, while mechanical stress and temperature fluctuations cause significant output drift and offset errors	This sensor technology supports BLDC motor commutation, automotive ABS speed and position sensing, overcurrent protection in EV and solar systems, and door/window security alarms.
MEMS (Micro-Electro-Mechanical Systems) [24,25,26]	10^−6^	MEMS sensors offer a low per-unit price, extreme miniaturization, high spatial resolution, and compatibility with thin-film microfabrication technologies, making them suitable for portable and integrated sensing applications.	Their performance may be limited by fabrication complexity, sensitivity to process variations, and reduced sensitivity compared with larger conventional magnetic sensing systems.	Common applications include consumer electronics, industrial automation, proximity sensing, displacement measurement, nondestructive testing, and microcrack detection.

## Data Availability

The design files for the proposed inductive MEMS sensor in GDS format are available online with prior access authorization at: https://drive.google.com/drive/folders/1-iYbg4tSpXM_P1pqegol7_aXuag5EknP?usp=drive_link, accessed on 15 March 2026.

## Abbreviations

The following abbreviations are used in this manuscript:

AC	Alternating Current
ADC	Analog-to-Digital Converter
AIN	Aluminum nitride
AMR	Anisotropic Magneto-Resistive
BEOL	Back-End-of-Line
b-LN	Bidomain Lithium Niobate
BLDC	Brushless Direct Current
CMOS	Complementary Metal-Oxide-Semiconductor
DC	Direct Current
EMC	Electromagnetic Compatibility
EMF	Electromagnetic Field
EV	Electric Vehicle
FGM	Fluxgate Magnetometer
FOD	Foreign Object Detection
GMR	Giant Magnetoresistance
HES	Hall Effect Sensor
ICNIRP	International Commission on Non-Ionizing Radiation Protection
LED	Light Emitting Diode
LFM	Lorentz-Force Magnetometers
ME	Magnetoelectric
MEMS	Micro Electro Mechanical Systems
MO	Magneto Optical
OFG	Orthogonal Fluxgate
PCB	Printed Circuit Board
SNR	Signal-to-Noise Ratio
SQUID	Superconducting Quantum Interference Devices
TMR	Tunneling Magneto Resistance
vHF	Vapor Hydrogen Fluoride
WPT	Wireless Power Transfer
